# A systematic review protocol of Tuina for children with acute bronchitis: A protocol for systematic review

**DOI:** 10.1097/MD.0000000000018899

**Published:** 2020-01-24

**Authors:** Huichao Feng, Jiao Rong, Ke Pei, Fushi Jing, Qian Zhuang, Tianjiao Lu, Fujie Jing, Jiguo Yang

**Affiliations:** a School of Acupuncture-Tuina; b School of Traditional Chinese Medicine, Shandong University of Traditional Chinese Medicine, Jinan; c Department of Rehabilitation, The People's Hospital of Juxian, Juxian, Shandong, China.

**Keywords:** acute bronchitis, children, protocol, systematic, Tuina

## Abstract

**Background:**

Acute bronchitis (AB) is a common cause of childhood morbidity. Tuina, a kind of Chinese massage, is frequently used for the treatment of AB in children by traditional Chinese medicine doctors. However, there is no relevant systematic review show its effectiveness and safety. The study aims to evaluate the effectiveness and safety of Tuina for children with AB.

**Methods:**

The following electronic databases will be searched from the respective dates of database inception to January 1st, 2020: The Cochrane Library, Web of Science, the World Health Organization International Clinical Trials Registry Platform, Springer, EMBASE, MEDLINE, China National Knowledge Infrastructure, the Chinese Biomedical Literature Database, Wanfang database, the Chinese Scientific Journal Database, and other sources. All published randomized controlled trials and blinded researches that are relevant to the subject of interest only will be contained. Two independent researchers will operate article retrieval, duplication removing, screening, quality evaluation, and data analyses by Review Manager (V.5.3.5). Meta-analyses, subgroup analysis and/or descriptive analysis will be performed based on the included data conditions.

**Results:**

High-quality synthesis and/or descriptive analysis of current evidence will be provided from the bronchitis severity score, symptom, and quality-of-life questionnaires, the questionnaire of clinical symptoms of cough and sputum, Patient Satisfaction Scale and adverse reactions.

**Conclusion:**

This study will provide the evidence of whether Tuina is an effective and safe intervention for children with AB.

**PROSPERO registration number:**

CRD42019140667.

## 1 Introduction

### 1.1 Description of the condition

Acute bronchitis (AB) is a lower respiratory tract infection characterized by cough, with or without sputum production, lasting several days to weeks. It is a self-limited disease dominated by viral infections. AB is a common disease that leads to pediatric outpatient treatment.^[1]^ As a whole, the incidence of AB in children continued to rise until the 1990s.^[2]^ The following factors is related to AB in children: air pollutants (eg, PM1, NO_2_, NOx, PM2.5, CO, daily PM10, sulfurous air pollution from volcanic eruption),^[3–6]^ season (eg, an annual peak in September–October),^[7]^ diurnal temperature range,^[8]^ genes (eg, repair gene XPC, xenobiotic metabolizing genes),^[9,10]^ coal home heating,^[11]^ family factors (eg, maternal smoking, low gestational age, low maternal age, older siblings).^[11,12]^ According to the World Health Organization, acute respiratory infections, including AB, are the leading cause of death in children under 5 years of age (except newborns) and rank at the sixth position in newborns.^[13]^

### 1.2 Description and function of intervention

Tuina (Chinese Massage), based on the viscera theory, meridian theory, 5 elements theory, is a kind of physical therapy of Traditional Chinese Medicine (TCM) with a long history. Tuina used in children dates back to the Qin and Han dynasties and prevails in the Ming dynasty.^[14]^ Tuina affects the physiology and pathology of the body by manipulation on specific parts of the body surface to prevent and cure diseases. Clinical literatures show that Tuina has therapeutic effect on AB and can reduce morbidity by improving immunity.^[15]^ TCM doctors often apply Tuina to treat AB, such as clearing Feijing, carrying inside Bagua, clearing Tianheshui, pushing Danzhong (RN17), rubbing Fenglong (ST40), rubbing Feishu (BL13), rubbing Dingchuan (EX-B1), rubbing Gaohuang (BL43).^[16–19]^

### 1.3 Why the review is important

AB increase visibly in terms of the global hospital incidence rate, and continue to be a serious health problem worldwide.^[20]^ They confer a significant economic burden.^[3,4,20]^ The disease may develop into chronic bronchitis, even further emphysema or pulmonary heart disease if it occurs repeatedly.^[4]^ The most common indications for antibiotic prescribing for children in 2012 were AB (25.6%), most of which were viral infections.^[21,22]^ Antimicrobial resistance is a serious threat to public health.^[23]^ In Chinese clinical trials, many treatments for children with AB have drawn the method of Tuina. Owing to nonstandard measurement, nonuniformed outcomes, subjectivity judgment, and other factors, the evidence was still limited. Furthermore, no relevant review or protocol has been published. As a consequence, it is necessary to conduct evidence-based review to evaluate the efficacy and safety of Tuina for AB in children. It is urgently needed to accomplish this review.

## 2 Methods

This systematic review has been registered in the PROSPERO network (No. CRD 42019140667). All steps of this systematic review will be performed according to the Cochrane Handbook (5.2.0).

### 2.1 Selection criteria

#### 2.1.1 Types of studies

Randomized controlled trial (RCT) and blinded research will be included. Published clinical trials that reported the efficacy and safety on Tuina for AB with children will be included. RCTs that involve at least 1 Tuina related treatment to AB, and 1 control treatment (or blank treatment) will be included. As there is a risk of interference with the outcome, nonrandomized controlled trials will be excluded. Studies of animal experiment, review, case report, and meta-analysis will be excluded.

#### 2.1.2 Types of patients

Patients who were diagnosed as AB, aged under 18 years, will be included, without limits on gender, race, nationality, and medical units.

#### 2.1.3 Types of interventions and comparisons

Interventions can be any type of Tuina: acupressure, pushing, kneading, pinching, rubbing, transiting, stroking, and chiropractics based on meridian-acupoint theory. Multiple control interventions will be included: no treatment, placebo and other interventions (eg, acupuncture, cupping therapy, drugs, and physical interventions, moxibustion). If its interventions and comparisons both contain Tuina, the study will be excluded. Interventions of Tuina combined with other therapies will be included, only if these combinations are compared to the other therapies semplice.

#### 2.1.4 Types of outcomes

Main outcomes will include the bronchitis severity score (BSS) and symptom and quality-of-life (QOL) questionnaires. BSS comprises the sum of 5 major symptom scores for AB: cough, sputum, dyspnoea, chest pain during coughing, and rales on auscultation. Each symptom is scored on a 4-point-scale (0 = absent, 1 = mild, 2 = moderate, 3 = severe, 4 = very severe), with a maximum total score of 20 points. QOL is a measure of cough frequency. Additional outcomes will include the questionnaire of clinical symptoms of cough and sputum, patient satisfaction scale, adverse reactions.

### 2.2 Search methods for identification of studies

#### 2.2.1 Electronic searches

The following electronic databases will be searched from the respective dates of database inception to January 1st, 2020: The Cochrane Library, Web of Science, the World Health Organization International Clinical Trials Registry Platform, Springer, EMBASE, MEDLINE, China National Knowledge Infrastructure, the Chinese Biomedical Literature Database, Wanfang database, the Chinese Scientific Journal Database, and other sources. All published RCTs about this subject will be included. Exemplary search strategy of MEDLINE is listed in Table 1, terms are conformed to medical subject heading. According to the different retrieval modes, keywords may combine with free words and comprehensive search will be performed.

**Table 1:**
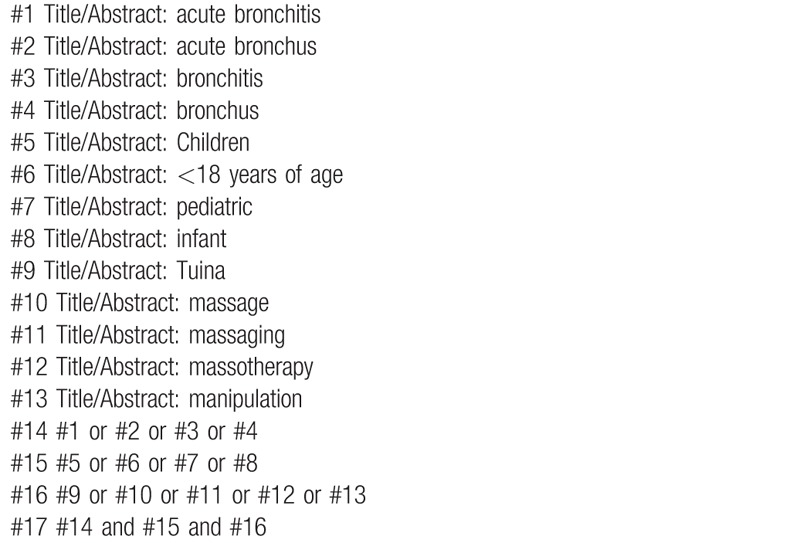
MEDLINE search strategy.

### 2.3 Data collection and analysis

#### 2.3.1 Selection of literature

**Figure 1:**
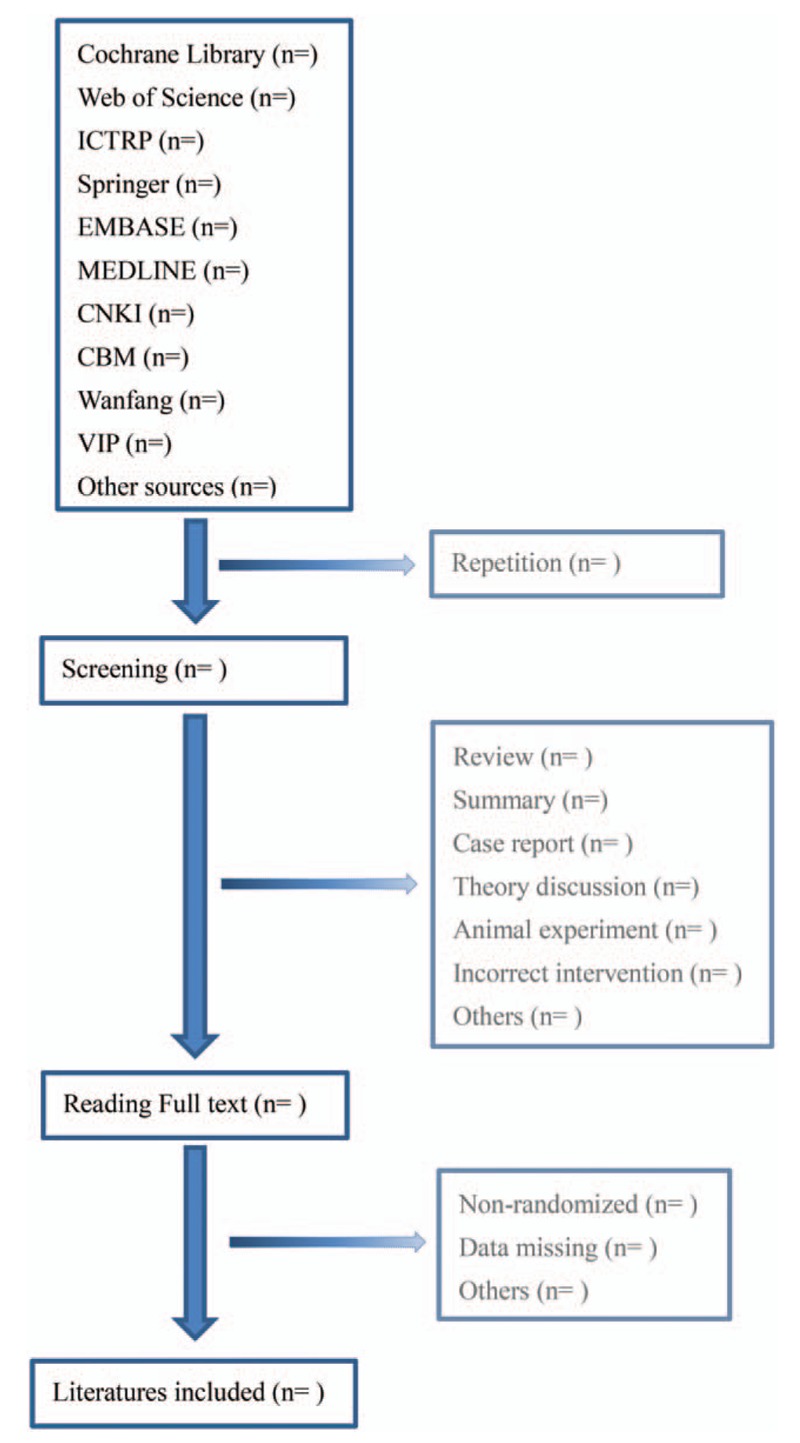
Flow diagram of literature retrieval.

Two authors (HF and JR) will select clinical trials conforming to inclusion criteria independently. After the articles are screened, disrelated, repetitive, nonstandard literatures will be excluded. Screening operation will be rendered in Figure 1. If the full literatures are unable to obtained or related data is incomplete, we will contact the corresponding author. Third-party experts will be consulted to determine the selection divergence.

#### 2.3.2 Assessment and quality of included literature

Two independent authors (KP and JR) will evaluate quality of included literature and assess the risk of bias by Review Manager (5.3.5) based on Cochrane Handbook (5.2.0). Quality will be assessed from 5 aspects: randomized method, allocation concealment, blinding methods (participants, personnel, outcome), completeness of outcome data, and selective reporting. Third-party experts will be consulted to determine the selection divergence.

#### 2.3.3 Data extraction

Two independent authors (HF and QZ) will extract the data from the articles selected for inclusion, and resolve differences in opinion through discussion with experts. Data will be recorded onto an electronic form, including categories for basic information about the studies (numbering, the first author's last name and the year the study was published, and the contact information for the corresponding author), the sample sizes and grouping methods used, participant characteristics including age and gender, expressed as mean additions and subtractions above and below standard deviation and the percentages, and details of the intervention methods involved, including treatment time, the selection of acupoints, treatment efficacy, treatment cycles, side effects, and follow-up.

#### 2.3.4 Measures of treatment effect

Two authors (HF and KP) will analysis independently and cross-check treatment effect by Review Manager 5.3.5. Risk ratio (RR) with 95% confidence intervals (CIs) will be adopted if dichotomous data exists. Continuous data will be presented by mean difference or standard mean difference with 95% CI. Other binary data will be changed into the RR form for analysis.

#### 2.3.5 Dealing with missing data

As the necessary data in the literature may be lack, we will contact the corresponding authors by email or other contacts. If the missing data is unavailable, we will analyze the existing data and suppose the missing data as random missing.

#### 2.3.6 Assessment of heterogeneity

The heterogeneity of studies will be evaluated by *Q*-test and *I*^2^ statistic with RevMan5.3.5. The following criteria will be used: *I*^*2*^ < 50% will be deemed as low heterogeneity; *I*^*2*^ between 50% and 75% will be considered as moderate heterogeneity; *I*^*2*^ > 75% will be considered as high heterogeneity.

#### 2.3.7 Assessment of reporting bias

Funnel plots will be created to assess the reporting bias. Dissymmetry funnel plot indicates high risk of reporting bias, while symmetric funnel plot indicates low risk.

#### 2.3.8 Data synthesis

A meta-analysis or descriptive analysis will be carried out based on measurement methods, intervention methods, heterogeneity levels, etc. If clinical and methodological heterogeneity are low, the fixed-effect model will be applied by merger analysis; the random-effects model will be applied by merger analysis when heterogeneity indicates a moderate level. If a significant level of heterogeneity is found, a descriptive analysis will be performed instead.

#### 2.3.9 Subgroup analysis

Subgroup analysis will be performed based on the findings from the data synthesis. If the heterogeneity is found to have been caused by particular features of the included studies (eg, the intervention methods [type, time, and cycle] and the measurement methods used in the clinical trials), subgroup analysis will be conducted relevant to these categories.

## 3 Discussion

AB has a high incidence in children. The antibiotics is not necessary in many cases. As a noninvasive external physiotherapy, Tuina is widely used for AB with children from ancient times to modern, and is popular in China with the characteristics of simple, convenience, low cost, and so on. In recent years, more and more clinical reports on the treatment of pediatric AB, but high quality trail is still insufficient. This review will begin when necessary trails are meeting. To give compelling evidence and better guide in clinic practice, all actions of this review will be performed according to Cochrane Handbook 5.2.0.

## Author contributions

**Conceptualization:** Huichao Feng, Fujie Jing, Ke Pei.

**Data curation:** Jiao Rong, Ke Pei, Fushi Jing.

**Investigation:** Qian Zhuang, Tianjiao Lu.

**Methodology:** Huichao Feng, Ke Pei.

**Supervision**: Huichao Feng, Jiao Rong.

**Validation:** Fujie Jing, Jiguo Yang.

**Visualization:** Huichao Feng.

**Writing – original draft:** Huichao Feng, Jiao Rong, Ke Pei.

**Writing – review & editing:** Fujie Jing, Jiguo Yang.

## References

[1] SW Y MJ P CK R HL301 in the treatment of acute bronchitis: a phase 2b, randomized, double-blind, placebocontrolled, multicenter study. [published online ahead of print, 2019 Apr 9]. Korean J Intern Med 2019;10.3904/kjim.2018.181. 10.3904/kjim.2018.181 PMC6960049 30962409

[2] Fleming DM Elliot AJ The management of acute bronchitis in children. Expert Opin Pharmaco 2007;8:415–26. 10.1517/14656566.8.4.415 17309336

[3] Nhung NTT Schindler C Dien TM Acute effects of ambient air pollution on lower respiratory infections in Hanoi children: an eight-year time series study. Environ Int 2018;110:139–48. 29128032 10.1016/j.envint.2017.10.024

[4] Bai L Su X Zhao D Exposure to traffic-related air pollution and acute bronchitis in children: season and age as modifiers. J Epidemiol Community Health 2018;72:426–33. 29440305 10.1136/jech-2017-209948

[5] D M J M DG F EH W research W-BRJBor. An observational study of PM10 and hospital admissions for acute exacerbations of chronic respiratory disease in Tasmania, Australia 1992–2002. BMJ Open Respir Res 2015;2 (1):e000063 Published 2015 Jan 7. 10.1136/bmjresp-2014-000063 PMC4289711 25593705

[6] Longo BM Yang W Acute bronchitis and volcanic air pollution: a community-based cohort study at Kilauea Volcano, Hawai’i, USA. J Toxicol Environ Health A 2008;71:1565–71. 18850456 10.1080/15287390802414117

[7] Ho NT Thompson C Nhan LNT Retrospective analysis assessing the spatial and temporal distribution of paediatric acute respiratory tract infections in Ho Chi Minh City, Vietnam. BMJ Open 2018;8:e016349. 10.1136/bmjopen-2017-016349 PMC5780701 29358416

[8] Xie MY Ni H Zhao DS Effect of diurnal temperature range on the outpatient visits for acute bronchitis in children: a time-series study in Hefei, China. Public Health 2017;144:103–8. 28274369 10.1016/j.puhe.2016.12.016

[9] Ghosh R Rossner P Honkova K Air pollution and childhood bronchitis: interaction with xenobiotic, immune regulatory and DNA repair genes. Environ Int 2016;87:94–100. 26655675 10.1016/j.envint.2015.10.002

[10] Ghosh R Topinka J Joad JP Air pollutants, genes and early childhood acute bronchitis. Mutat Res 2013;749:80–6. 23648357 10.1016/j.mrfmmm.2013.04.001

[11] RJ B, I H-P, M D, et al. Coal home heating and environmental tobacco smoke in relation to lower respiratory illness in Czech children, from birth to 3 years of age. 2006; 114(7):1126–1132. 10.1289/ehp.8501 PMC1513340 16835069

[12] Braback L Bjor O Nordahl G Early determinants of first hospital admissions for asthma and acute bronchitis among Swedish children. Acta Paediatr 2003;92:27–33. 12650295 10.1111/j.1651-2227.2003.tb00464.x

[13] UN Inter-agency Group for Child Mortality Estimates. Causes of Child Death. The Media Center of World Health Organization Network, November 2019. Available at: https://www.who.int/data/gho/data/themes/topics/indicator-groups/indicator-group-details/GHO/causes-of-child-death.

[14] Zhang X Guo T Zhu B Pediatric Tuina for promoting growth and development of preterm infants: a protocol for the systematic review of randomized controlled trail. Medicine (Baltimore) 2018;97:e0574. 29718853 10.1097/MD.0000000000010574 PMC6392971

[15] Liu Y Research progress of external therapy in the treatment of acute bronchitis in children. Chinese Community Doctors 2016;32:7–9.

[16] Xu X The Clinical Research on Acute Bronchitis of Food Retention Phlegmy Heat Type in Children with Manipulation. Changqing District, Jinan, Shandong, China: Shandong University of Traditional Chinese Medicine, 4655 University Road; 2015.

[17] Zhao L Clinical observation on treatment of children acute bronchitis with phlegm-heat obstructing lung by massage plus feishu(Bl13) cupping. Chin Arch Tradit Chin Med 2018;36:1199–201.

[18] Xin M Treat 94 cases of acute bronchitis in children by Tuina with medium of Tankejing powder. Hunan J Tradit Chin Med 2012;28:100–1.

[19] Xin M Shi C Jiang X A clinical study on the treatment of acute bronchitis in children by massage on the point of chest and back. J Sichuan Tradit Chin Med 2008 116–7.

[20] Ben Ayed H Yaich S Ben Jmaa M Pediatric respiratory tract diseases: chronological trends and perspectives. Pediatr Int 2018;60:76–82. 28891268 10.1111/ped.13418

[21] Karinauske E Kasciuskeviciute S Morkuniene V Antibiotic prescribing trends in a pediatric population in Lithuania in 2003-2012: observational study. Medicine (Baltimore) 2019;98:e17220. 31725600 10.1097/MD.0000000000017220 PMC6867790

[22] Morgan JR Carey KM Barlam TF Inappropriate antibiotic prescribing for acute bronchitis in children and impact on subsequent episodes of care and treatment. Pediatr Infect Dis J 2019;38:271–4. 29794648 10.1097/INF.0000000000002117 PMC7918285

[23] Hay AD Redmond NM Turnbull S Development and internal validation of a clinical rule to improve antibiotic use in children presenting to primary care with acute respiratory tract infection and cough: a prognostic cohort study. Lancet Respir Med 2016;4:902–10. 27594440 10.1016/S2213-2600(16)30223-5 PMC5080970

